# Effect of Boar Sperm Proteins and Quality Changes on Field Fertility

**DOI:** 10.3390/ani11061813

**Published:** 2021-06-17

**Authors:** Ilias Michos, Maria Tsantarliotou, Constantin M. Boscos, Georgios Tsousis, Athina Basioura, Eleni D. Tzika, Panagiotis D. Tassis, Aristotelis G. Lymberopoulos, Ioannis A. Tsakmakidis

**Affiliations:** 1 Faculty of Health Sciences, School of Veterinary Medicine, Aristotle University of Thessaloniki, 54627 Thessaloniki, Greece; imichos84@hotmail.com (I.M.); mtsant@vet.auth.gr (M.T.); pboscos@vet.auth.gr (C.M.B.); tsousis@vet.auth.gr (G.T.); basioura@vet.auth.gr (A.B.); eltzika@vet.auth.gr (E.D.T.); ptassis@vet.auth.gr (P.D.T.); 2 Laboratory of Farm Animal Reproduction & Animal Breeding, Department of Agriculture, School of Geotechnical Sciences, International Hellenic University, 57001 Thessaloniki, Greece; alymperopoulos@ihu.gr

**Keywords:** artificial insemination, boar, field fertility, proteins, sperm

## Abstract

**Simple Summary:**

Artificial insemination with extended liquid boar semen is widely used in the swine industry. The identification of the relationship between boar sperm characteristics and fertility could be of substantial importance to reproduction management. This study evaluated the relationship between boar sperm characteristics and sperm/seminal plasma proteins with main parameters of field fertility. Immotile spermatozoa and spermatozoa with biochemically active plasma membranes affected the number of live-born piglets and litter size of ≥12 piglets. The proteins osteopontin 70 and glutathione peroxidase 5, both separately and in combination, affected the farrowing rate. The combination of immotile sperm and protein osteopontin 70 explained the variation regarding litter size with ≥12 piglets. In conclusion, the evaluation of semen quality variables combined with the evaluation of specific sperm or seminal plasma proteins could provide useful information on in vivo fertilizing capacity of semen doses.

**Abstract:**

This study aimed to evaluate boar sperm characteristics and proteins, in relation to their importance regarding in vivo fertility. Sixty-five ejaculates were used and 468 sows (parity ≥ 2) were inseminated. Sperm CASA kinetics, morphology, viability, DNA fragmentation, mitochondrial membrane potential, sperm membrane biochemical activity (HOST) and sperm proteins (Heat Shock Protein 90-HSP90, glutathione peroxidase-5-GPX5, Osteopontin 70-OPN70) were assessed and related to field fertility (number of live-born piglets—NLBP, litter size ≥ 12 piglets—LS, farrowing rate—FR). Statistical analysis was conducted with simple and multiple regression models. Simple regression analysis showed that immotile sperm (IM) significantly affected the NLBP and LS, explaining 6.7% and 6.5% of their variation, respectively. The HOST positive spermatozoa significantly affected the NLBP and LS, explaining 24.5% and 7.8% of their variation, respectively. Similarly, sperm with activated mitochondria significantly affected the NLBP, explaining 13.5% of its variation. Moreover, the OPN70 affected LS and FR, explaining 7.5% and 10.8% of their variation, respectively. Sperm GPX5 protein affected FR, explaining 6.7% of its variation. Multiple regression analysis showed that the combination of IM and/OPN70 explains 13.0% of the variation regarding LS, and the combination of GPX5 and OPN70 explains 13.6% of the variation regarding FR. In conclusion, the estimation of parameters IM, membrane biochemical activity, mitochondrial membrane potential, OPN and GPX5 can provide useful information regarding semen doses for field fertility.

## 1. Introduction

Boars play a key role in pig farms’ productivity and economic efficacy. In the swine industry, the fertilization of sows is exclusively achieved by artificial insemination (AI) with extended liquid boar semen. This choice has contributed to faster genetic improvement and reduced production costs, while the boars’ impact on the reproductive outcome has substantially increased [1]. Numerous researchers have attempted to correlate the qualitative sperm characteristics with fertility, deriving conflicting results. Initial difficulties were due to a lack of objectivity and repeatability, attributable to the use of subjective sperm assessment techniques [2]. However, the development of computer assisted sperm analyzing systems (CASA) reduced the influence of human factors, thus improving the accuracy and objectivity of the measurements [3].

Today, the farrowing rates by artificial insemination exceed 90%, and the number of live-born piglets is higher than 12. The achieved litter size of 12–14 piglets increases the reproductivity and reduces the early culling of the sows, improving the income of the pig farms [4].

Obviously, fertility is a complex process, influenced by both boar and other factors regarding animal management. The cellular factors associated with the fertilization failure of spermatozoa are not always clearly explained, with sperm and seminal plasma proteins needing to be involved in research studies to clarify this issue. Reactive oxygen species (ROS) are beneficial for sperm hyperactivation and acrosome reaction [5]. Nonetheless, the imbalance between ROS and the antioxidant mechanisms, which leads to oxidative stress, can be destructive, causing cell death and reducing fertilizing capacity [6]. Glutathione peroxidase (GPx) regulates small changes in the concentration of H2O2 or other derivatives. Alvarez and Storey [7] highlighted its protective role against the loss of sperm motility due to peroxidation of membrane lipids. In humans, non-expression of GPx in sperm causes infertility [8]. The family of the glutathione peroxidase is categorized in five classes (GPX1-GPX5), based on their sequence and location. GPX5 is a specialized protein, which inhibits the early acrosome’s response when the spermatozoa are located in the epididymis’ tail. Moreover, Kilian et al. [9] identified one sperm plasma protein of 55 kDa molecular weight which is positively related with bull fertility. This protein was later identified as osteopontin (OPN) [10]. It is believed that OPN attaches to spermatozoa during ejaculation, and it remains attached until they reach the isthmus of the fallopian tubes [11]. In camels, OPN has been positively correlated with fertility [12]. Additionally, it has been reported to improve the effectiveness of swine [13], buffalo [14] and cattle [15] in vitro fertilization (IVF). In boar spermatozoa, the Western Blot method identified at least two forms of OPN with different molecular weights, i.e., different isoform versions, possibly with different functions [16]. Furthermore, the body responds to heat stress by increasing the synthesis of a group of proteins called Heat Shock Proteins (HSPs) [17]. This group of proteins makes sperm more resistant to temperature changes. The chaperone HSP90 is the most studied Heat Shock Protein [18] which increases sperm thermal resistance and protects spermatozoa from apoptosis and oxidative stress [19,20]. It has been found that lower expression of HSP90 is related to a greater cold stress sensitivity of sperm [21] and to a subsequent reduction of motility [22].

The aim of this study was first to evaluate boar sperm variables, as well as to detect and quantify selected sperm and seminal plasma proteins (HSP90, GPX5 and OPN). Secondly, we aimed to investigate the possible relation between the examined sperm parameters and proteins (HSP90, GPX5 and OPN) regarding field fertility.

## 2. Materials and Methods

The study was approved by the Research Committee of the School of Veterinary Medicine, Aristotle University of Thessaloniki, Greece (code 19/05/06/12). No animals were handled. All operations took place according to the University’s guidelines for animal research.

The chemicals and reagents used in this study were purchased from Sigma Aldrich (Seelze, Germany), unless otherwise specified.

### 2.1. Animals and Semen Collection

Eighteen (18) crossbred boars, kept in a farrow-to-finish farm under veterinary monitoring, were used in this study. All animals involved were vaccinated against major swine pathogens and dewormed according to the farm’s regular preventive scheme. In total, 65 ejaculations (3–4 per boar) were collected, processed, and then used to inseminate 468 sows of parity ≥ 2 with conventional AI throughout a year. From each ejaculate, an average of 7.2 sows were inseminated. For each ejaculate, the fertility outcomes (live-born piglets, proportion of litter sizes with ≥12 piglets and farrowing rate) derived from its use were recorded. The average values were then calculated and used for further analysis.

The boars and sows were housed under intensive farming conditions, where a balanced diet and ad libitum water were provided [23]. The boars were housed in individual pens under controlled environmental conditions. The whole ejaculate was collected with the “gloved hand technique”. The gelatinous phase of each ejaculate was removed after filtration and discarded. All collected samples fulfilled the quality criteria for AI semen dose preparation (at least 300 × 10^6^ sperm/mL, 70% motile spermatozoa, and 80% spermatozoa with normal morphology).

After that, the semen was divided into two aliquots, and different processes were followed according to the respective laboratory tests. The first aliquot was processed for proteomic analysis, and the second one was used for the assessment of sperm quality and functionality variables.

### 2.2. Preparation of Sperm and Seminal Plasma Proteins for Proteomic Analysis

In the beginning, the first portion was mixed with a protease inhibitor cocktail (4-(2-aminoethyl) benzenesulfonyl fluoride, pepstatin A, E-64, leupeptin, bestatin, aprotinin) and centrifuged (640× *g*; 15 min; 17 °C) to separate sperm cells and seminal plasma [24] (González-Cadavid et al., 2014). Seminal plasma was centrifuged for a second time (10,000× *g*; 15 min; 17 °C), and finally the supernatant was stored at −80 °C until further examination.

The sperm pellet was diluted with Phosphate Buffer Solution (PBS) to a concentration of 1.5 × 10^9^ spermatozoa/mL. The diluted sperm was firstly centrifuged (640× *g*; 3 min; 17 °C), then washed with 10 mL PBS and finally re-centrifuged (800× *g*; 5 min; 17 °C) [25]. The last sperm pellet produced was resuspended with HAM F-10 1X (Thermofisher^®^ Scientific, Regensburg, Germany), and the sample was once again centrifuged, whereas the supernatant was removed. Following this, the spermatozoa were solubilized in NP-40 lysis buffer (50 mM Tris–HCL pH 7.4, 250 mM NaCl, 5 mM EDTA, 1% Glycerol, 0.5% NP-40, 1 mM DTT, 1 mM PMSF 100 mM, 1 × protease inhibitor cocktail (Roche, Athens, Greece)) and the aliquot was stored at −80 °C until further analysis.

### 2.3. Semen Sample Processing for the Performance of Sperm Analysis

The second aliquot of collected semen was extended with a commercial extender (M III^®^, Minitube, Tiefenbach, Germany) to a final concentration of 30 × 10^6^ spermatozoa/mL, divided to insemination doses and stored in the farm’s storage facilities at 17 °C. One semen dose per boar was used, transported to the laboratory (within an hour) inside an air-conditioned isothermal box (Minitube, Tiefenbach, Germany) adjusted to 17 °C for further analysis.

### 2.4. Performance of Artificial Insemination, Data Recording

Estrus detection was carried out every 12 h, using a boar and the criterion of the standing reflex. At 12 and 24 h after the detection of estrus, two conventional inseminations were performed (volume 100 mL, concentration of 30 × 10^6^ spermatozoa/mL) in 468 sows with parity 2 ≤ 5. Data were collected four months after the end of the study (number of live-born piglets, proportion of litter sizes with ≥12 piglets and farrowing rate). The parameter “litter size with ≥12 piglets” was chosen since it is the lower desired limit to reduce culling of sows and increase their use for reproduction in the farm [4].

### 2.5. Assessment of Sperm Motility and Kinetics

Sperm motility was evaluated using computer-assisted semen analysis (CASA-Sperm Class Analyser^®^, Microptic S.L., Automatic Diagnostic Systems, Barcelona, Spain) and a microscope (100×; AXIO Scope A1, Zeiss, Jena, Germany) accomplished with a heating stage. Ten (10) μL of each semen sample was placed on the preheated Makler chamber (Makler^®^ counting chamber, 10 μm deep, Sefi Medical Instruments, Haifa, Israel) at 37 °C, and triple assessment of each semen sample was applied.

The following CASA motility parameters and kinetics were estimated: (i) total motility %; (ii) progressive spermatozoa %; (iii) rapid spermatozoa % (45 < rapid μm/s); (iv) VCL—curvilinear velocity (μm/s); (v) VSL—straight line velocity (μm/s); (vi) VAP—average path velocity (μm/s); (vii) ALH—amplitude of lateral head displacement (μm); (viii) BCF—beat/cross-frequency (Hz); (ix) LIN—linearity (VSL/VCL × 100); (x) STR—straightness (VSL/VAP × 100); (xi) WOB—wobble (VAP/VCL × 100); (xii) hyperactivated spermatozoa % (VSL > 97 μm/sec, ALH > 3.5 μm, LIN < 0.32). The following configuration of the CASA software was used: eight fields and >500 spermatozoa, 25 frames/s, region of particle control 10–18 μm, progressive movement of >45% of the parameter STR, circumferential movement <50% of parameter LIN, depth of field 10 μm and temperature of the microscope plate at 37 °C. The objects incorrectly identified as spermatozoa were manually removed before the final analysis by the Sperm Class Analyzer software (SCA^®^ v.5.2.0.0., Microptic S.L., Barcelona, Spain).

### 2.6. Assessment of Sperm Morphology

Sperm morphology was evaluated by the SpermBlue staining method (SpermBlue^®^ 08029, Microptic S.L., Barcelona, Spain) according to the manufacturer’s instructions. Spermatozoa were assessed microscopically (×400) and classified as normal or abnormal (head (including integrity of acrosome membrane), neck, tail, cytoplasmic droplets). Totally, 200 spermatozoa per sample were counted, and the results were expressed as % ratio.

### 2.7. Assessment of Sperm Viability

Sperm viability was assessed using double fluorescent stain calcein-AM (C-AM; 1 mmol/L) and propidium iodide (PI; 0.75 mmol/L). Briefly, 100 μL of semen was mixed with 5 μL of C-AM and 1 μL of PI and incubated at 37 °C in the dark for 15 min. The evaluation took place under a fluorescence microscope (×400). Spermatozoa with intact plasma membranes fluoresce green, while the dead spermatozoa fluoresce red. In total, 200 spermatozoa per slide were evaluated, and the results were expressed as the percentage of live spermatozoa per sample.

### 2.8. Assessment of HOST

A slight modification of Vazquez et al. [26] took place to perform the Hypo-Osmotic Swelling Test. The HOST solution was produced with fructose (75 mmol/L) and sodium citrate (32 mmol/L). The osmolality was set to 150 mOsm using a cryoscopic osmometer (OSMOMAT^®^ 030, Gonotec, Berlin, Germany). Briefly, 100 μL of semen sample was mixed with 1 mL of HOST solution and incubated for 1 h at 37 °C. Spermatozoa with functional plasma membranes present swollen tails. In total, 200 spermatozoa per slide were evaluated (×400). The results were expressed as the percentage of spermatozoa with swollen tails.

### 2.9. Assessment of Mitochondrial Membrane Potential

Rhodamine 123 (Rh123; 0.01 mgr/mL)/PI (0.75 mmol/L) dual fluorescent staining was performed to assess mitochondrial membrane potential [27]. Digital photos were immediately taken under a fluorescent microscope and a total of 200 spermatozoa were scored. The percentage of live sperm with functional mitochondria was identified by R123 high fluorescence and no PI fluorescence, and results were expressed as % ratio.

### 2.10. Assessment of DNA Integrity

The Acridine Orange Test (AOT) was applied to determine the sperm DNA integrity [28]. In total, 200 spermatozoa were counted using a fluorescence microscope (×1000). The results were reported as the percentage of spermatozoa with DNA fragmentation.

### 2.11. Sperm and Seminal Plasma Protein Analysis

Total spermatozoa and seminal plasma protein quantification was performed according to Bradford [29], and bovine albumin was used for the construction of the standard curve.

The sperm pellet was thrice centrifuged (800× *g*; 1 min) and resuspended with PBS. Sperm proteins were lysed in 5 × SDS loading buffer (Tris 250 mM, SDS 10%, Glycerol 50%, β-Mercaptoethanol 15%), then boiled for 5 min and loaded onto 12% slab gels for electrophoresis (SDS-PAGE). Gel electrophoresis was run for 1 h at 50 mA/gel constant current (Mini Protean^®^ 3 CellBio-Rad, Berkley, CA, USA). Afterwards, the proteins were transferred onto nitrocellulose membranes (GE Healthcare) under 50 mA/300 V for 1 h (PowerPac 1000, Bio-Rad). Then, membranes were thrice washed in TBST (20 mM Tris, 500 mM NaCl, 0.05% Tween; pH 7.5) and incubated with blocking solution (5% skimmed milk powder in PBST) for 1 h. Subsequently, the membranes were washed for 5 min in TBST and incubated for 2 h with the primary antibodies (rabbit polyclonal anti-HSP 90 (AP-22747PU-N; Acris; diluted 1:1000 with TTBS), rabbit polyclonal anti-GPX5 (18731-1-AP; Proteintech Europe; diluted 1:500) and rabbit polyclonal anti- beta actin (ab8227, Abcam)). Membranes were thrice washed and incubated with a horseradish peroxidase-conjugated polyclonal goat anti-rabbit immunoglobin (SC-2004; Santa Cruz; 1:2000). Membranes were developed with Pierce™ ECL Plus Western Blotting Substrate (Thermoscientific, Rockford, IL, USA) and scanned with Typhoon FLA 7000 (GE Healthcare). Protein levels were expressed as “band volume”; that is, the total signal intensity measured inside the boundary of a band in pixel intensity units.

**Figure 1:**
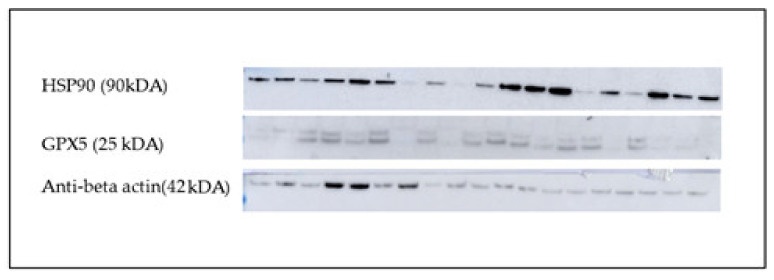
Indicative band patterns of spermatozoa HSP90 and GPX5. Actin was used as an internal standard.

**Figure 2:**
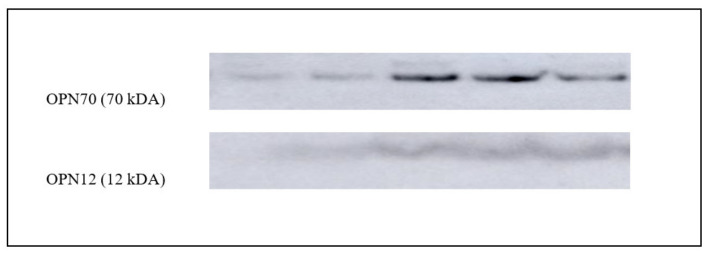
Indicative band patterns of seminal plasma OPN. Two reactive areas were located at 70 kDa and 12 kDa.

**Figure 3:**
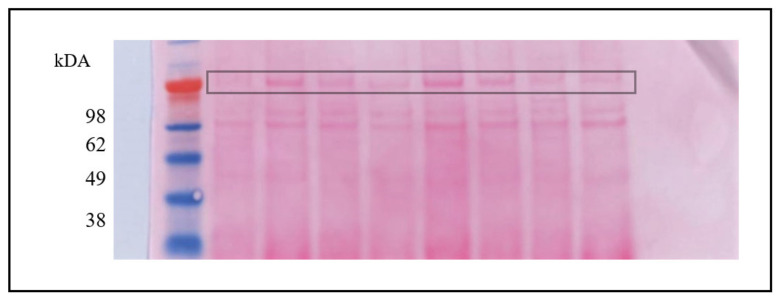
Nitrocellulose blot stained with Ponceau S. The protein band that was used as OPN standard is highlighted.

The quantification of protein bands was performed with ImageJ software (v.1.8.0), and actin was used as a normalizing factor (Figure 1). The ratio of protein bands and actin gives the level of proteins [25]. Each examination was performed three times, and then the mean was used. According to Valencia et al. [18], SDS-PAGE, Western Blot and quantification of seminal plasma proteins were likewise carried out. For the detection of osteopontin, anti-OPN antibodies (GTX 37582; GeneTextech; diluted 1:200) were used (Figure 2). In addition, a non-specific band from Ponceau S staining was used as a normalizing factor (Figure 3). Both bands were quantified with ImageJ (v.1.8.0), and their ratio was used for graphical presentation. The mean of three distinct estimations for each seminal plasma sample was considered.

### 2.12. Statistical Analysis

The software package SAS (Statistical Analysis System Institute Inc., version 9.3, Cary, NC, USA) was used for calculations. All variables were graphically illustrated, and the Shapiro–Wilk test was used to test for normality of the data. Regarding the parameters OPN70 and OPN12, two and four outliers were omitted from further analysis, respectively. Simple regression analysis between sperm parameters and fertility traits (i.e., mean of live-born piglets, mean percent of farrowings with litter size equal to or more than 12 piglets per insemination and mean farrowing rate per ejaculate) was performed. Variables that had a significant effect were further analyzed with multiple regression analysis. Multiple regression analysis was applied with a forward selection process. Level of entry for a variable was set to 0.20, and level of stay in the model was set to 0.05. The adjusted coefficient of determination was used to measure the goodness of fit of the model. Data are presented as mean ± standard deviation. *p* < 0.05 was considered significant.

## 3. Results

The reproductive data after the insemination of 468 sows, the CASA analysis, and the quality variables of the 65 boar semen samples used in AI are presented in Table 1, Table 2 and Table 3, respectively. The obtained results are within the normal range for pig farm productivity and the approved quality limits for the use of extended fresh boar semen in AI.

The effect of sperm kinetics on reproductive outcomes is presented in Table 4. Live-born piglets and litter sizes with ≥12 piglets showed significant relationships (*p* < 0.05) with sperm motility parameters. Specifically, the percentage of immotile spermatozoa explained 6.7% (*p* = 0.04) of the variation of live-born piglets and 6.5% (*p* = 0.04) of the variation of litter sizes with ≥12 piglets.

The associations between semen quality variables and reproductive data are listed in Table 5. The highest coefficient of determination of the present study was observed between live-born piglets and the HOST. The percentage of HOST (+) spermatozoa explained 24.5% (*p* = 0.0001) of the variation of live-born piglets and 7.8% (*p* = 0.03) of the variation of litter sizes with ≥12 piglets. The variation of live-born piglets was additionally explained by the percentage of activated mitochondria (*p* = 0.01) by 13.5%.

The effect of the proteins HSP90, GPX5 and OPN (Figure S1) on the reproductive parameters is listed in Table 6. Osteopontin (OPN) was found to be associated with fertility parameters. Specifically, OPN70, as recorded in Western Blot, explained 7.5% (*p* = 0.03) of the variation of litter sizes with ≥12 piglets. Regarding the farrowing rates, the analysis of the results showed that GPX5 explained 6.7% (*p* = 0.04) and OPN70 explained 10.8% (*p* = 0.009) of its variation.

Furthermore, multiple regression analysis of the results demonstrated that the resistance of sperm to stress in a sub-osmotic solution (HOST) and the percentage of activated mitochondria had a statistically significant effect on the number of live births (*p* = 0.009, *p* = 0.03, respectively). However, the total variation explained did not increase significantly with the addition of the second variable (percentage of activated mitochondria), compared to the value derived from simple regression. In contrast, the combination of immotile spermatozoa and OPN70 explained 13.0% of the variation regarding litter size with ≥12 piglets (adjusted coefficient of determination). Additionally, regarding the farrowing rate, GPX5 and OPN70 together explained 13.6% of its variation.

## 4. Discussion

In the present study, the relationship between boar sperm quality variables and field fertility was investigated over the course of a year. Moreover, specific sperm and seminal plasma proteins were detected and related with the artificial insemination outcome. Protein selection was based on previous reports regarding their potential role in fertility and sperm protection against stress [16,21,30].

Sperm motility is one of the most studied parameters, as it has been related to the energy condition of spermatozoa [31]. The thorough analysis of the kinematics with CASA systems created new scientific data with conflicting results. Holt et al. [32] positively correlated VAP, VSL, VCL, ALH and BCF with fertility. In contrast, around the same time, Popwell and Flowers [33] were unable to correlate motility with field fertility, while Flowers [34] found that sperm motility values > 60% were not reliably correlated with farrowing rate and litter size. In subsequent studies, Gadea et al. [35] found a low correlation between sperm motility and farrowing rate. This finding was reinforced by Vyt et al. [36], in terms of total and live-born piglets. In their two studies, McPherson et al. [37,38] initially found a positive correlation only between the BCF parameter and the number of stillborn piglets, and then between the VAP/VCL parameters and the increased return to estrus rates after insemination. Summarizing the above, Amann and Waberski [39] reported that the parameters of motility, as derived from the use of CASA analyzer, cannot be used to predict boar sperm fertilizing capacity, while there are large variations in the results. The results of the present study are in agreement with this opinion, as only a relationship between the percentage of immotile sperm and the number of live-born piglets (r^2^ = 0.067, *p* = 0.04), as well as the farrowings with ≥12 piglets (r^2^ = 0.065, *p* = 0.04), were observed. Our findings coincide with those of Broekhuijse et al. [40], who, in a large-scale study, concluded that only 6% of total fertility variation can be explained by boar sperm factors. In fact, they positively correlated progressive motility, VCL and BCF with the farrowing rate, as well as the total motility and VAP with the live-born piglets, but negatively correlated ALH and VSL with the total piglets born. The above-mentioned researchers used a Leja chamber in their estimates, while a Makler chamber was used in the present study. Many researchers have stated that the CASA outcomes could be influenced by the analysis’ chambers [41,42,43]. In addition, in the present study, low-motility ejaculates were initially rejected because they could not be used for artificial insemination. The general difficulty of finding a strong association between sperm motility and fertility parameters may lie in the fact that passive sperm transport also plays an important role. According to Langendijk et al. [44], after insemination, the transport of sperm from the entrance of the cervix to the fallopian tubes is affected by the condition of the genital epithelium, the contractions of the myometrium, and the degree of the female olfactory, visual, and auditory stimulation from the presence of a boar.

Furthermore, Tsakmakidis et al. [28] found that the combination of sperm morphology and the stability of sperm genetic material strongly correlated with farrowing rates, while Myromslien et al. [45] positively correlated sperm motility, morphology, and DNA fragmentation with the number of piglets born. In the present study, no relationship was found between field fertility and DNA fragmentation, but there were no ejaculates with significant percentages of DNA damage to safely document any result. This point of view is supported by the results of Boe-Hansen et al. [46], who concluded that DNA damage rates of 2.1–3% decrease reproductive performance, as well as the results of Roca et al. [47], who found that DNA fragmentation > 20% results in 2–3 piglets less per farrowing. Moreover, it should not be overlooked that DNA damage is associated with boar individuality and is often a random finding in pig farms [48].

Sperm behavior under osmotic stress (HOS test) proved to be a satisfactory prognostic indicator, as we found a significant relationship with the percentage of live-born piglets (*p* = 0.0001). In fact, the effect of this relationship reached 24.5%, and from the regression equation (data not shown), the parameter estimate was 0.05, which can be interpreted as 0.05 more piglets per farrowing for every 1% increase in the values of HOST. A positive yet not as strong correlation also emerged between HOST positive spermatozoa and litter sizes with ≥12 piglets (r^2^ = 0.078, *p* = 0.03). According to Vazquez et al. [26], the HOS test can determine the percentage of biochemically active sperm. The findings of the present study agree with those of Yeste et al. [49]; sperm tests in sub-osmotic and hyper-osmotic solutions are highly related to the expected litter sizes, thus they can be used as alternatives to other conventional tests. Gadea and Matas [50] also found an association between HOST outcome and fertility; however, this was not greater compared to other common sperm assessment methods.

Examination of mitochondrial function by double-staining Rhodamine/PI showed that the percentage of sperm with activated mitochondria was positively related with the number of live born piglets (*p* = 0.01), explaining 13.5% of its variation. No relationship was detected between mitochondrial function and the other recorded fertility parameters. Our findings agree with those of Schulze et al. [51], who found that the percentage of sperm with active mitochondria on the 2nd day of boar sperm storage is positively related to the number of piglets born. Using different fluorescent staining, Huo et al. [52] concluded that mitochondrial function can be positively correlated with the boar sperm viability and acrosome integrity.

The lack of an association between sperm viability and the examined fertility variables could be attributed to the fact that, in our study, only ejaculates of acceptable quality were evaluated and used to perform AI. Similar conclusions were reached by Gadea et al. [35] and McPherson et al. [37], who used eosin-nigrosine and did not find any relationship between viability, litter sizes and live-born piglets. Viability highlights the integrity of spermatozoa membranes but not their functionality, which makes it a less reliable predictor of fertility. Therefore, the evaluation of viability is more useful for the initial exclusion of ejaculates with a high percentage of dead spermatozoa.

Extensive research has already been carried out concerning the correlation of fertility with the proteomic profile of boar sperm, whilst a new effort to investigate the lipidomic profile of boar seminal plasma has arisen to enforce knowledge about this topic [53]. Osteopontin (OPN) has been identified in both female [54] and male reproductive systems [55], but its action has not yet been sufficiently clarified [56]. It is known that OPN is secreted by the auxiliary glands and, during ejaculation, binds to spermatozoa, forming a complex that interacts with the zona pellucida [57]. Osteopontin has been positively associated with fertility in stallions [58] and bulls [10]. Four times higher concentrations of OPN were found in high-fertility bulls compared to low-fertility bulls [59]. In pigs, the addition of OPN to the IVF medium resulted in better early embryo formation, reducing apoptosis and fragmentation [13,60]. Susan et al. [16] identified OPN in three protein bands (OPN9, OPN12 and OPN70), yet they did not manage to correlate them with in vivo fertility. In the present study, only OPN12 and OPN70 were identified, possibly because polyclonal antibodies of a different source were used. The analysis of the data revealed for the first time the existence of a significant relationship between OPN70 and large litter sizes (≥12 piglets, r^2^ = 0.075), and between OPN70 and farrowing rates (r^2^ = 0.108). However, in both cases, this relationship was negative, and higher values of OPN70 seem to suppress fertility outcomes. It is thought that these findings may stimulate further investigation of OPN as a predictor of sperm fertilizing capacity.

Chaperone HSP90 is involved in sperm resistance mechanisms to cold stress [20,21]; therefore, it was selected as a prognostic indicator of the fertility of boar sperm in the present study. On the other hand, Valencia et al. [18] showed that increased concentrations of HSP90 in boar sperm were related to limited resistance to the freezing process. The results of our study did not show any relationship between HSP90 and boar fertility. This could be explained by the fact that fresh, gently manipulated sperm without heat-exposure was used to inseminate the sows. Calle-Guisado et al. [61] identified a beneficial effect of HSP90 on sperm motility and mitochondrial function only after exposure to thermal stress. Moreover, Huang et al. [22] detected some correlation between HSP70 (related to HSP90) and sperm quality characteristics, raising questions and stimulating further investigation about the importance of the HS protein family in the fertilization process.

Glutathione-5 (GPX5) peroxidase was selected for evaluation in the present study, since the GPx protein family is a main defense sperm system against free radicals [62]. GPX5 is secreted by the final layer of the epididymis [62] and binds to the surface of spermatozoa, protecting them from the destructive effects of peroxides as they pass through the epididymis [63]. The analysis of the results showed a significant and positive relationship between the farrowing rates (*p* = 0.04) and the concentration of GPX5, as this enzyme could explain 6.7% of their variation. Our results agree with those of Barranco et al. [64], who evaluated the presence of GPX5 in seminal plasma and found that boars with high GPX5 levels demonstrated more advanced farrowing rates and litter sizes compared to boars with low GPX5 levels. The results from both studies strengthen the view that GPX5 could be examined as a possible biomarker for boar fertility.

## 5. Conclusions

In conclusion, the HOS test seems to be the most reliable indicator for the interpretation of the variation of live-born piglets after AI in pig farms. Among other boar sperm assessment tests, percentages of immotile sperm, and spermatozoa with activated mitochondria may also explain the variation of live-born piglets. The variation of litter sizes with >12 piglets can be explained to some extent by the percentage of immotile spermatozoa and the concentration of OPN70. Concentrations of GPX5 and OPN70 enzymes are associated to some extent with the variation of farrowing rates.

## Figures and Tables

**Table 1:** Reproductive data of 468 inseminated sows.

Reproductive Data	Mean ± SD
Farrowing rate (%)	82.8 ± 24.6
Farrowings with ≥12 piglets (%)	45.8 ± 27.7
Total piglets born	11.7 ± 1.2
Live-born piglets	11.6 ± 1.2
Dead and stillborn piglets	0.1 ± 0.2

**Table 2:** Sperm kinetic parameters after CASA analysis of boar semen samples (n = 65).

Variables	Mean ± SD
Total motility (%)	88.58 ± 9.4
Progressive motility (%)	48.66 ± 14.7
Immotile spermatozoa (%)	8.86 ± 0.9
Rapid spermatozoa (%)	51.17 ± 21.7
Medium spermatozoa (%)	19.72 ± 8.9
Slow spermatozoa (%)	17.68 ± 9.9
VCL (μm/sec)	62.64 ± 27.7
VSL (μm/sec)	23.13 ± 7.5
VAP (μm/sec)	39.88 ± 12.3
LIN (%)	39.61 ± 11.9
STR (%)	58.25 ± 9.6
WOB (%)	67.49 ± 13.5
ALH (μm)	2.17 ± 0.5
BCF (Hz)	9.42 ± 4.0
Hyperactivated spermatozoa (%)	0.18 ± 0.1

VCL: curvilinear velocity (μm/sec); VSL: straight line velocity (μm/sec); VAP: average path velocity (μm/sec); LIN: linearity (VSL/VCL × 100); STR: straightness (VSL/VAP × 100); WOB: wobble (%); ALH: amplitude of lateral head displacement (μm); BCF: beat/cross-frequency (Hz).

**Table 3:** Quality semen variables and proteins of sperm and seminal plasma of the 65 boar samples.

Variables	Mean ± SEM
Normal morphology (%)	85.98 ± 10.4
Viability (%)	82.81 ± 7.7
HOST + spermatozoa (%)	38.90 ± 12.5
SAMM (%)	84.79 ± 7.4
DNA fragmentation (%)	0.17 ± 0.3
HSP90	2.46 ± 3.2
GPX5	3.43 ± 3.4
OPN70	3.65 ± 5.4
OPN12	4.29 ± 6.1

HOST: Hypo-Osmotic Swelling Test; SAMM: spermatozoa with active mitochondrial membranes; HSP90: heat shock protein 90; GPX5: glutathione peroxidase class 5; OPN70: osteopontin 70; OPN12: osteopontin 12.

**Table 4:** Effect of boar sperm kinetics on reproductive outcomes.

	Live-Born Piglets	Litter Size ≥ 12 Piglets (%)	Farrowing Rate (%)
Variables	r^2^	r^2^	r^2^
Immotile spermatozoa	0.067 *	0.065 *	0.002
Progressive motility	0.034	0.015	0.004
Rapid spermatozoa	0.006	0.01	0.005
Medium spermatozoa	0.016	0.012	0.005
Slow spermatozoa	0.001	0.005	0.002
VCL	0.000	0.000	0.001
VSL	0.000	0.000	0.008
VAP	0.000	0.001	0.000
LIN	0.000	0.000	0.034
STR	0.001	0.001	0.027
WOB	0.000	0.001	0.017
ALH	0.003	0.046	0.005
BCF	0.000	0.013	0.001
Hyperactivated spermatozoa	0.017	0.048	0.011

* Parameters showing a significant effect (*p* < 0.05) VCL: curvilinear velocity (μm/s); VSL: straight line velocity (μm/s); VAP: average path velocity (μm/s); LIN: linearity (VSL/VCL × 100); STR: straightness (VSL/VAP × 100); WOB: wobble (%); ALH: amplitude of lateral head displacement (μm); BCF: beat/cross-frequency (Hz).

**Table 5:** Effect of boar semen quality variables on reproductive outcomes.

	Live-Born Piglets	Litter Size ≥ 12 Piglets (%)	Farrowing Rate (%)
Variables	r^2^	r^2^	r^2^
Normal morphology	0.016	0.001	0.000
Viability	0.025	0.013	0.003
HOST + spermatozoa	0.245 *	0.078 *	0.019
SAMM	0.135 *	0.030	0.002
DNA Fragmentation	0.005	0.025	0.015

* Parameters showing a significant effect (*p* < 0.05). HOST: Hypo-Osmotic Swelling Test; SAMM: spermatozoa with active mitochondrial membranes.

**Table 6:** Effect of sperm/seminal plasma proteins on reproductive outcomes.

	Live-Born Piglets	Litter Size ≥ 12 Piglets (%)	Farrowing Rate (%)
Variables	r^2^	r^2^	r^2^
HSP90	0.043	0.015	0.033
GPX5	0.006	0.000	0.067 *
OPN70	0.008	0.075 *	0.108 *
OPN12	0.050	0.050	0.023

* Parameters showing a significant effect (*p* < 0.05). HSP90: heat shock protein 90; GPX5: glutathione peroxidase class 5; OPN70: osteopontin 70; OPN12: osteopontin 12.

## Data Availability

Data sharing not applicable.

## Supplementary Materials

The following are available online at https://www.mdpi.com/article/10.3390/ani11061813/s1, Figure S1: Original western blot figures for Figure 1, Figure 2 and Figure 3.
