# Supplementary material for: Effect of Boar Sperm Proteins and Quality Changes on Field Fertility
Source: Animals (Basel). 2021 Jun 17;11(6):1813. doi: 10.3390/ani11061813 (PMC8234339; doi:10.3390/ani11061813)
Supplement: Supplementary file 1 [file animals-11-01813-s001.zip › animals-1259014-SI.pptx]

## Slide 1
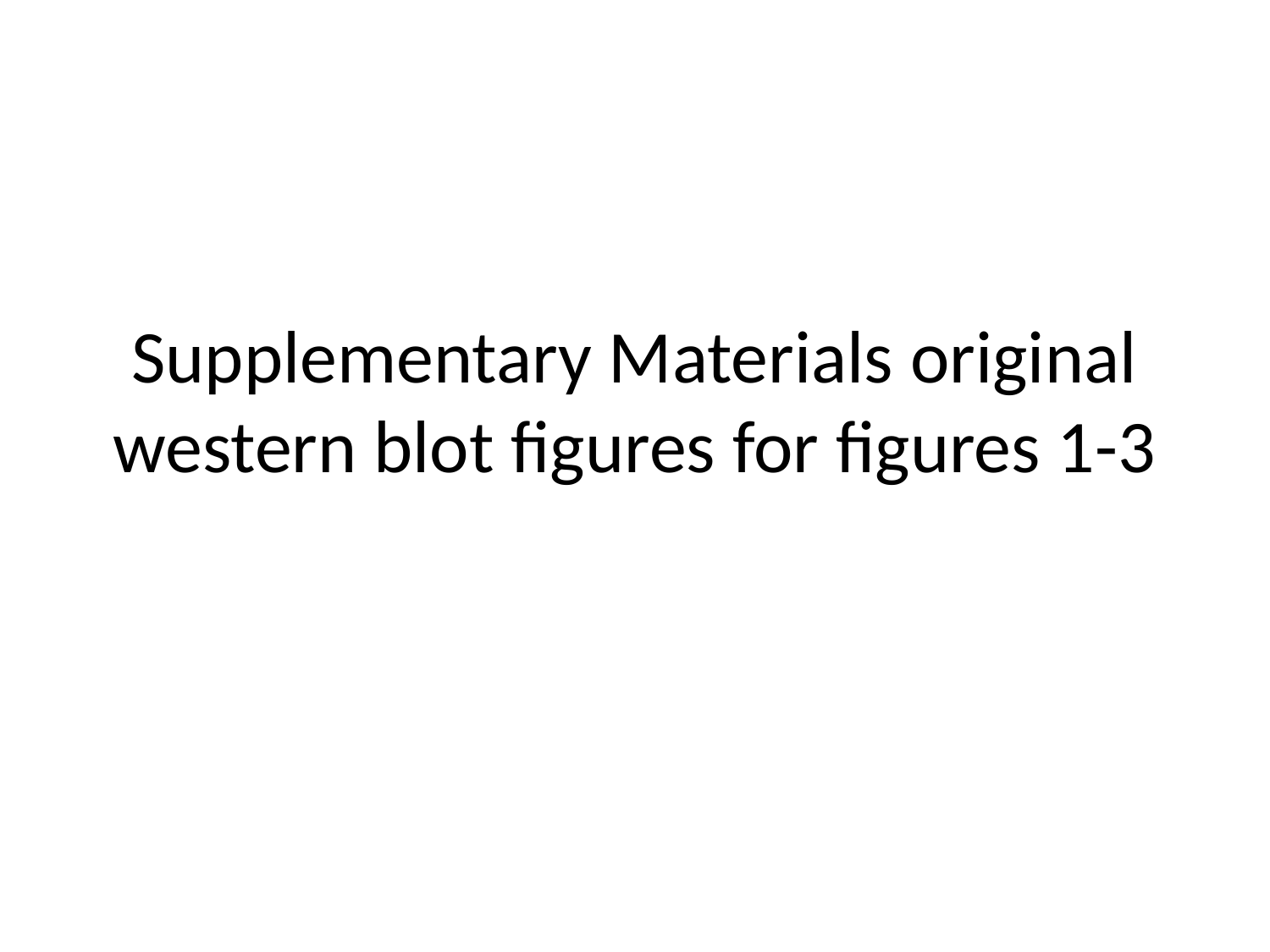

# Supplementary Materials original western blot figures for figures 1-3

## Slide 2
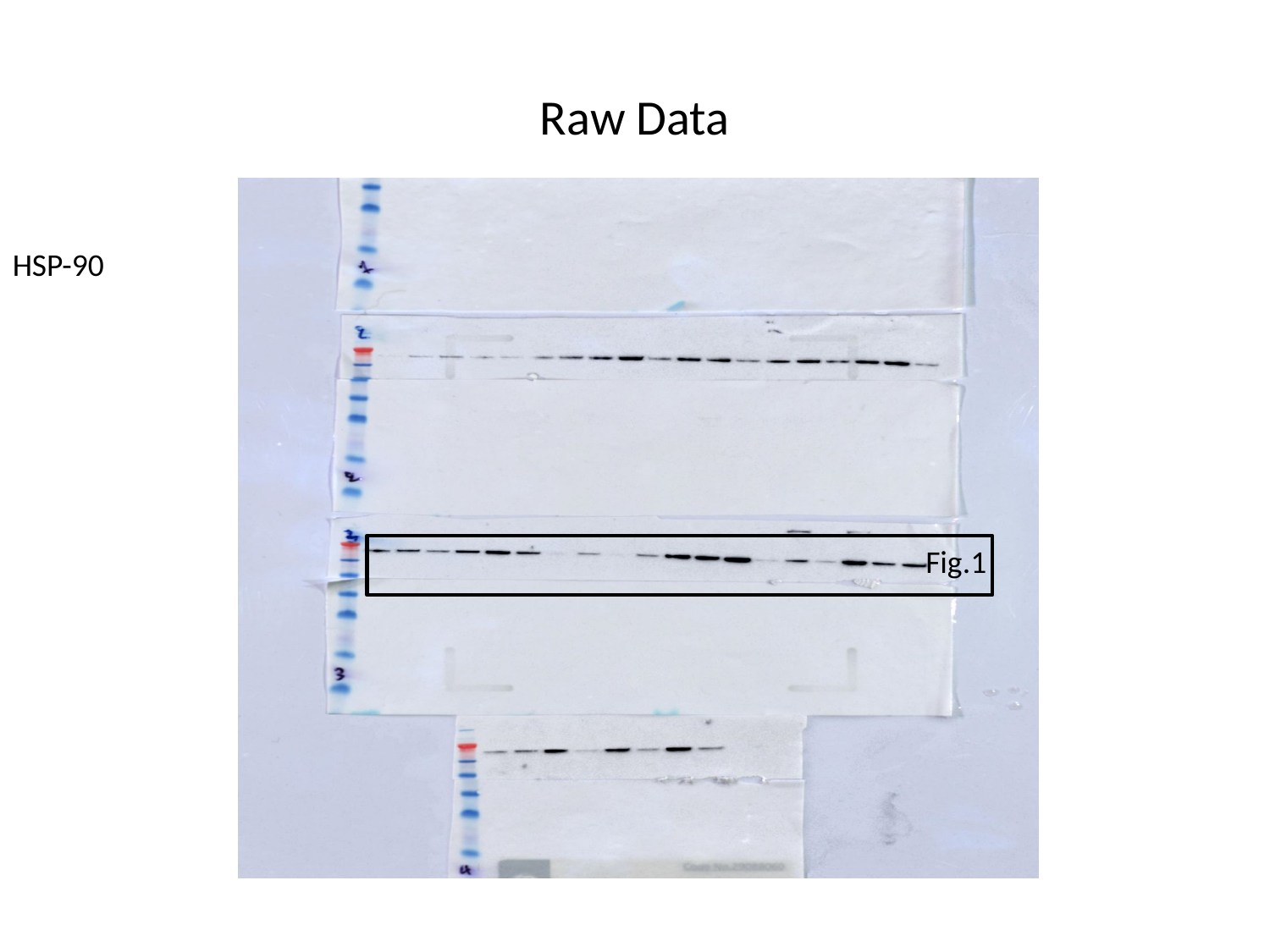

Raw Data
HSP-90
Fig.1

## Slide 3
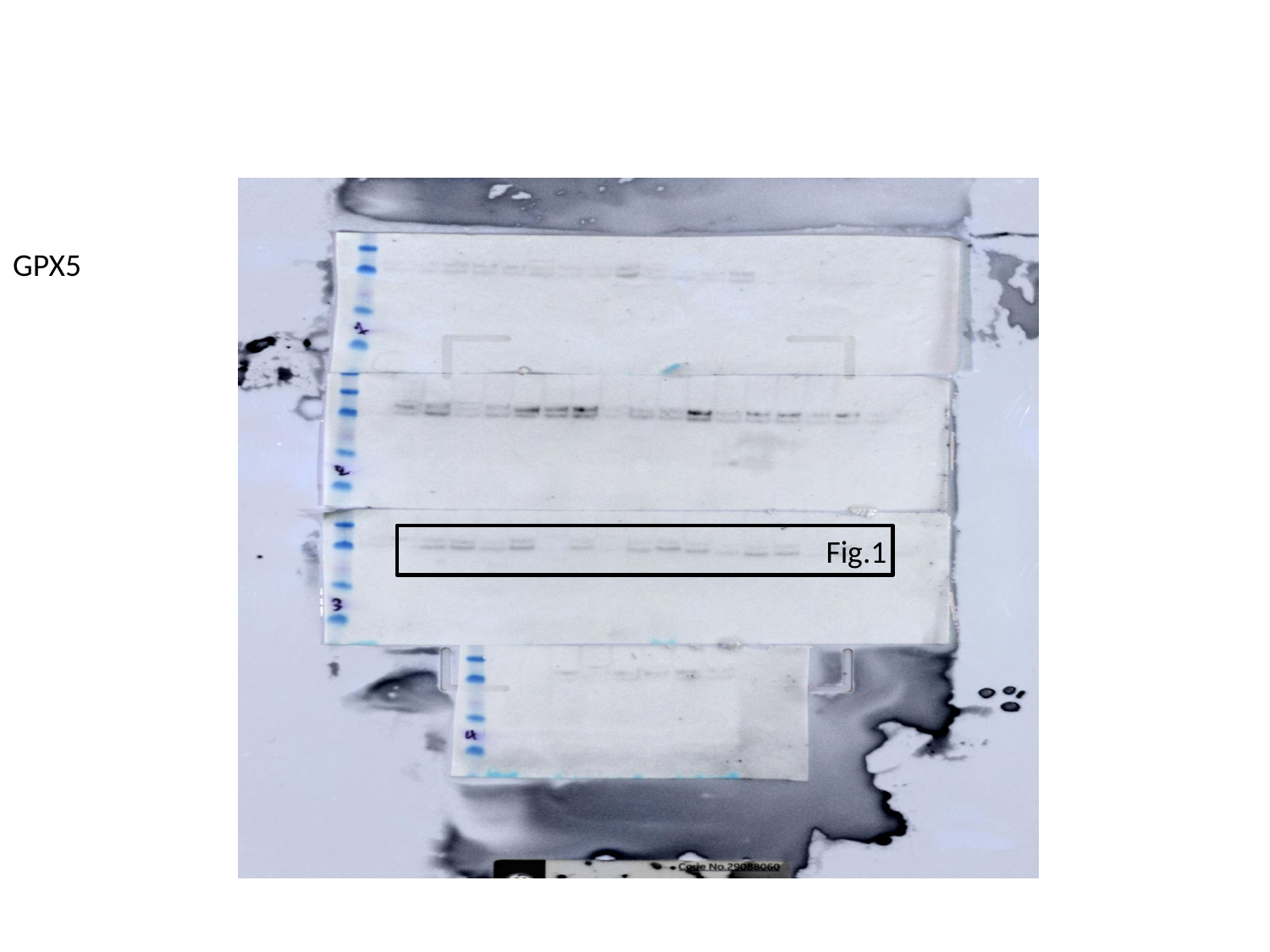

GPX5
Fig.1

## Slide 4
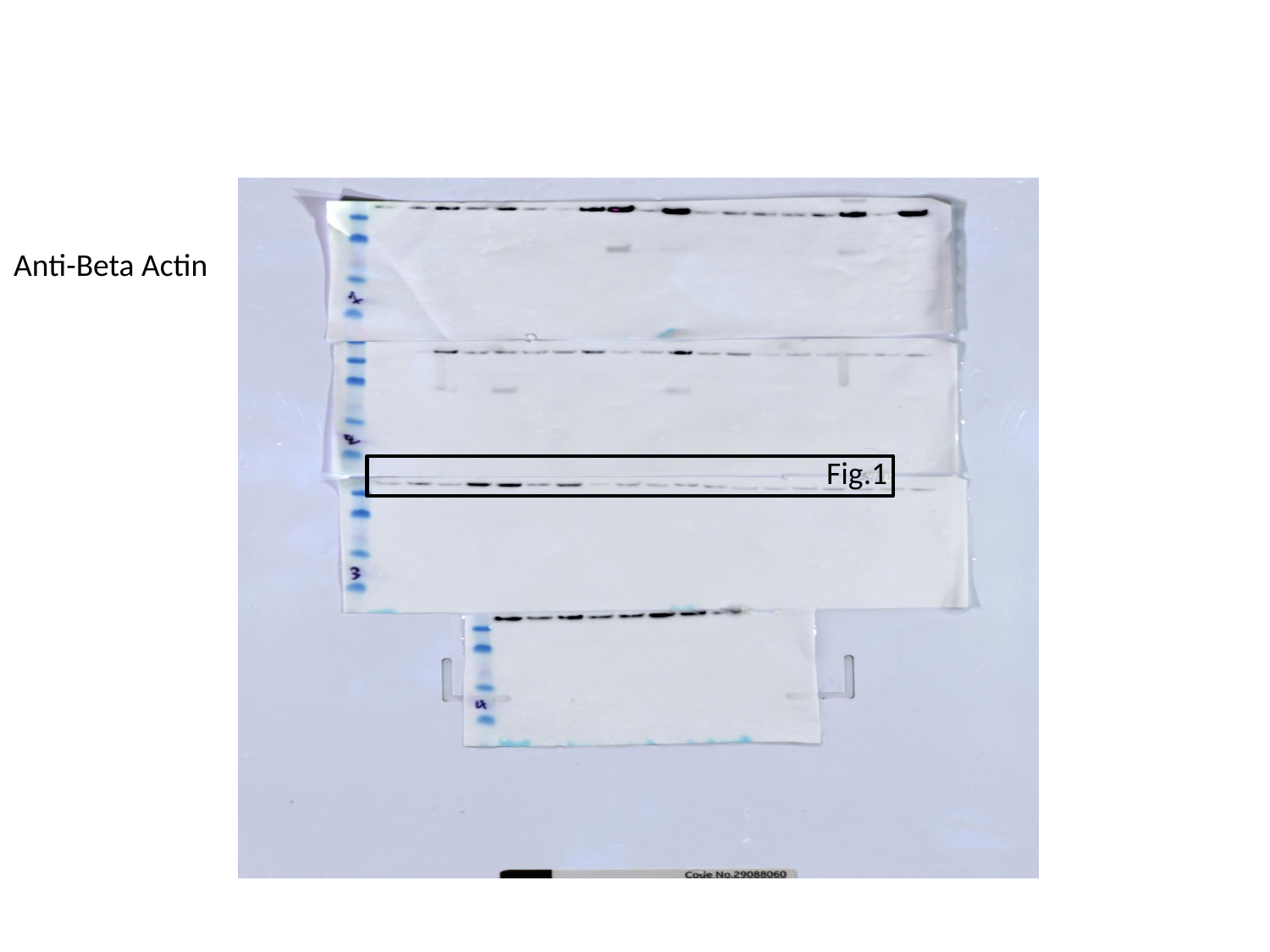

Anti-Beta Actin
Fig.1

## Slide 5
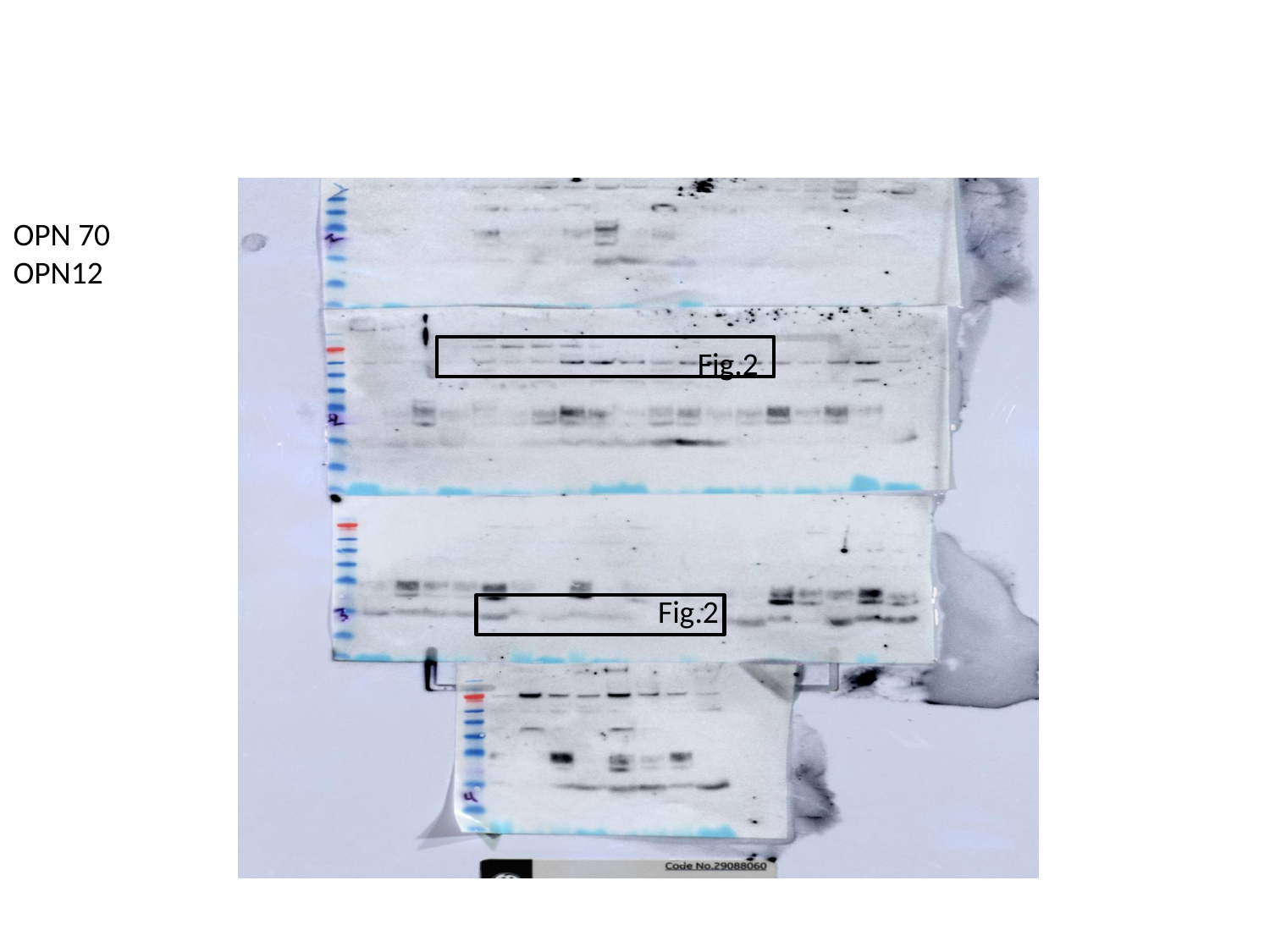

OPN 70
OPN12
Fig.2
Fig.2

## Slide 6
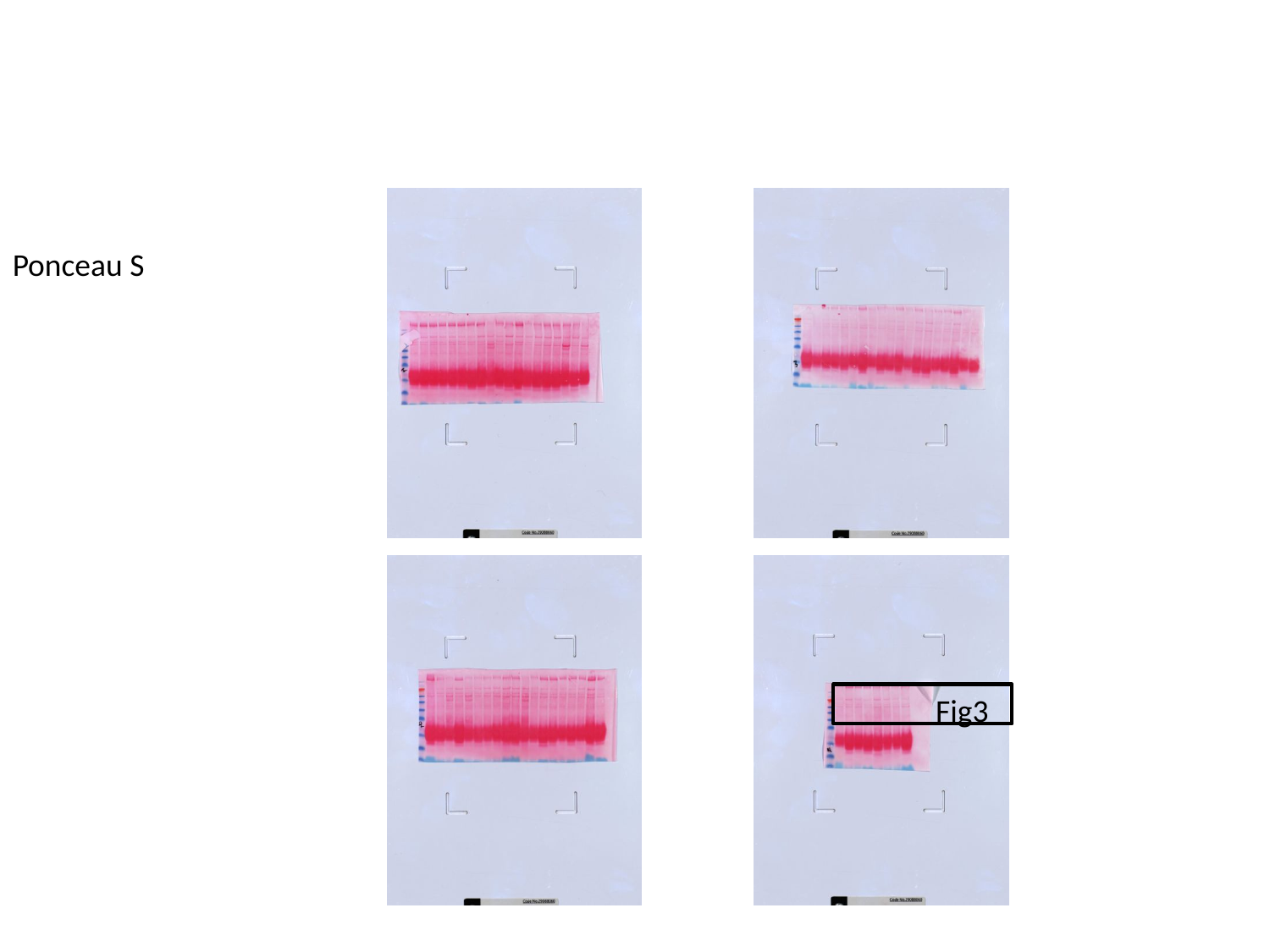

Ponceau S
Fig3
